# AKAP4 is a circulating biomarker for non-small cell lung cancer

**DOI:** 10.18632/oncotarget.3946

**Published:** 2015-05-13

**Authors:** Kiranmai Gumireddy, Anping Li, David H. Chang, Qin Liu, Andrew V. Kossenkov, Jinchun Yan, Robert J. Korst, Brian T. Nam, Hua Xu, Lin Zhang, Ganepola A.P. Ganepola, Louise C. Showe, Qihong Huang

**Affiliations:** ^1^ The Wistar Institute Cancer Center, Philadelphia, PA 19104, USA; ^2^ Center for Cancer Research and Genomic Medicine, The Valley Hospital, Paramus, NJ 07652, USA; ^3^ University of Washington Medical Center, Seattle, WA 98195, USA; ^4^ Department of Surgery, The Valley Hospital, Ridgewood, NJ 07450, USA; ^5^ Helen F. Graham Cancer Center & Research Institute, Christiana Care Health System, Newark, DE 19713, USA; ^6^ Department of Urology, Tongji Hospital, Tongji Medical College, Huazhong University of Sciences and Technology, Wuhan 430030, China; ^7^ Center for Research on Early Detection and Cure of Ovarian Cancer, University of Pennsylvania, Philadelphia, PA 19104, USA

**Keywords:** circulating biomarker, cancer

## Abstract

Cancer testis antigens (CTAs) are widely expressed in tumor tissues, circulating tumor cells (CTCs) and in cancer derived exosomes that are frequently engulfed by lymphoid cells. To determine whether tumor derived CTA mRNAs could be detected in RNA from purified peripheral blood mononuclear cells (PBMC) of non-small cell lung cancer (NSCLC) patients, we assayed for the expression of 116 CTAs in PBMC RNA in a discovery set and identified AKAP4 as a potential NSCLC biomarker. We validated AKAP4 as a highly accurate biomarker in a cohort of 264 NSCLCs and 135 controls from 2 different sites including a subset of controls with high risk lung nodules. When all (264) lung cancers were compared with all (135) controls the area under the ROC curve (AUC) was 0.9714. When 136 stage I NSCLC lung cancers are compared with all controls the AUC is 0.9795 and when all lung cancer patients were compared to 27 controls with histologically confirmed benign lung nodules, a comparison of significant clinical importance, the AUC was 0.9825. AKAP4 expression increases significantly with tumor stage, but independent of age, gender, smoking history or cancer subtype. Follow-up studies in a small number of resected NSCLC patients revealed a decrease of AKAP4 expression post-surgical resection that remained low in patients in remission and increased with tumor recurrence. AKAP4 is a highly accurate biomarker for the detection of early stage lung cancer.

## INTRODUCTION

Lung cancer is the leading cause of cancer deaths in both men and women in the US and results in more deaths globally than breast, prostate and colon cancers combined [1]. While the five year survival rate for early stage non-small cell lung cancer (NSCLC) is above 50% it is less than 5% in patients with metastatic disease [2]. Clearly, early detection can save lives, but accurate screening tests for high-risk individuals are still lacking. Although low dose computed tomography (LDCT) has been successfully used for screening in high-risk populations [3–5], multiple negative factors are associated with recurrent LDCT screening including false-positives and negatives, unnecessary invasive procedures, radiation exposure, global availability of the technology and cost [5]. Although several non-invasive tests for lung cancer using body fluids such as blood, urine or sputum are under investigation, none is currently in use.

When LDCT is used for screening, up to 51% of smokers 50 years or older are diagnosed with pulmonary nodules [6]. However only a small fraction of the nodules detected are subsequently diagnosed as lung cancer [7–9]. In cases where it is difficult to differentiate malignant from benign nodules, it is recommended that patients with these indeterminate nodules be followed with serial LDCT, which increases radiation exposure and financial cost. A simple, inexpensive blood test that differentiates malignant from benign nodules would fill an important clinical need.

Cancer/testis (CT) genes are a family of genes that are normally expressed in germ cells and trophoblasts, but aberrantly expressed in cancer cells [10, 11]. Approximately half of the Cancer/testis genes are on the X chromosome [11] and play an important role in germ cell development [10, 11]. Aberrant CT gene expression in cancer cells elicits immune responses [10, 11] with autoantibodies being detected in cancer patients [12, 13]. In this report we describe a highly accurate PCR based test for the presence of mRNA for the CTA gene AKAP4 in PBMC derived RNA that distinguishes patients with NSCLC from current and ex-smokers including those with histologically confirmed benign lung nodules.

## RESULTS

### Identification of AKAP4 as a lung cancer biomarker in peripheral blood mononuclear cell (PBMC) preparations

**Figure 1:**
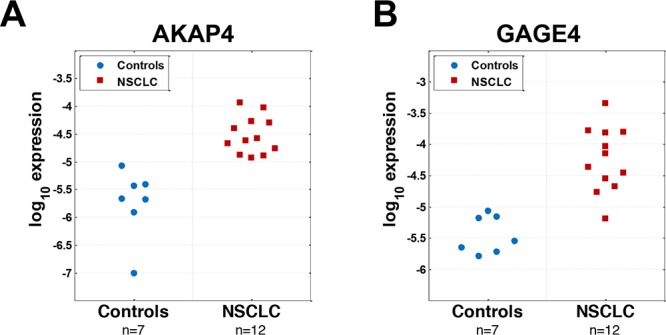
Unbiased nested PCR screening of 130 CTAs identifies AKAP4 and GAGE4 as potential candidates for NSCLC diagnosis based on a small discovery set of samples **A.** AKAP4 demonstrates perfect separation of samples from NSCLC and control groups based on its expression **B.** only 1 cancer sample was misclassified by expression of GAGE4.

It has been suggested that CT genes on human X chromosome (CT-X) may serve as markers of cancer cells with stem-cell-like properties, which may circulate in blood [10, 11]. The restricted expression of CT-X genes in most normal cells and their aberrant expression in many cancers including NCSLCs make them attractive potential biomarkers [14–16]. To address the potential utility of CTA genes as blood based biomarkers for the detection of NSCLCs, we designed unique PCR primers for 116 of the 130 CT genes on the X chromosome [17] (sequence similarities prevented the selection of specific primers for 14 CT-X genes). We applied nested PCR as the detection method because it was likely mRNAs would be present at low levels in the PBMC fraction being tested. We first tested whether any of the 116 CT-X genes were differentially expressed in PBMC derived RNA from a discovery set of 12 NSCLC lung cancer patients and 7 control patients with smoking related benign lung diseases including COPD and/or benign granulomatous inflammation. Four of the controls had histologically confirmed benign lung nodules. These highly characterized samples were a part of a previously described microarray study to develop blood based biomarkers for NSCLC [18, 19]. Based on results from the discovery set, we selected two candidate CTX genes that distinguished NSCLC from benign lung disease in this data set with the best accuracy for further analysis on a larger independent sample set. Expression of AKAP4 perfectly separated cancer and control groups (Figure 1A), while GAGE4 (Figure 1B) misclassified only one NSCLC sample, the best results among all 116 candidates tested (data not shown).

### Validation of AKAP4 mRNA as a lung cancer biomarker

**Figure 2:**
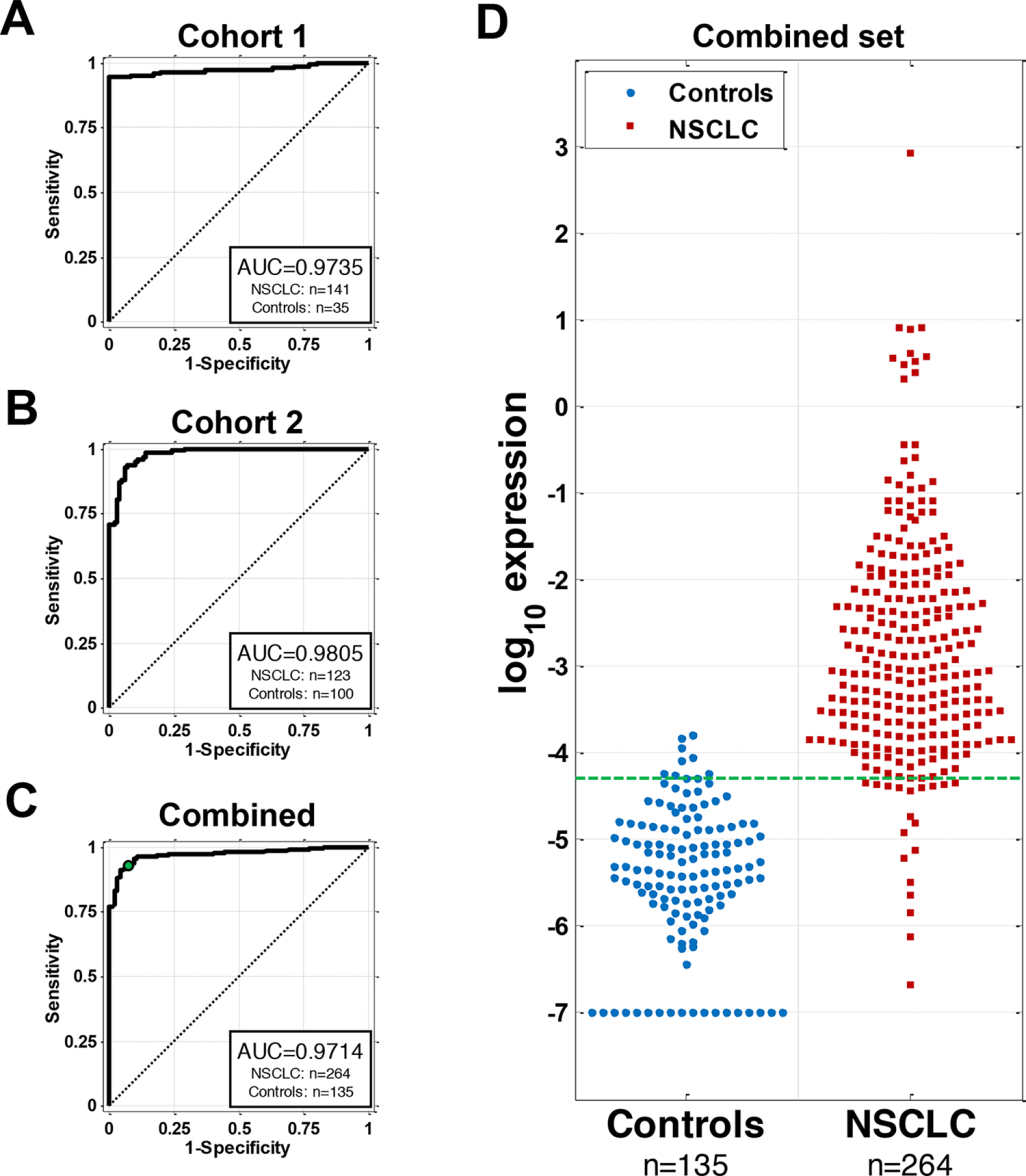
AKAP4 serves as a circulating biomarker for NSCLC in two cohorts of NSCLC patients and controls **A.** ROC curve of AKAP4 expression in the PBMC samples from 141 NSCLC patients and 35 patients with benign lung diseases. **B.** AKAP4 is validated as a circulating biomarker for NSCLC in the second independent patient cohort. ROC curve of AKAP4 expression in the PBMC samples from 123 NSCLC patients and 100 controls is shown. **C.** ROC curve for combined set of cohorts 1 and 2. The dot indicates the performance corresponding to selected optimal cutpoint. **D.** Distribution of AKAP4 expression levels in NSCLC and controls. Green dotted line indicates cutpoint optimized for a balanced sensitivity (92.8%) and specificity (92.6%).

To further confirm the potential utility of AKAP4 and GAGE4 as NSCLC biomarkers, we analyzed an additional cohort of 141 NSCLC patients and 35 control patients with benign lung diseases which did not include any of the discovery set samples (Table 1). Twenty four of the 35 controls had lung nodules confirmed as benign by biopsy (18). Although the accuracy for both AKAP4 and GAGE4 were essentially identical on the small data set, the performance of AKAP4 expression on the larger data set was significantly higher than GAGE4 with an AUC for AKAP4 in this comparison of 0.9735 (Figure 2A) and an AUC for GAGE4 of 0.7149 (Supplementary Figure 1). After testing and finding that combination of AKAP4 and GAGE4 expression did not improve overall prediction (data not shown), and because of its low AUC in this larger sample set, we did not include GAGE4 in the analysis going forward.

**Table 1:** Demographics of patients for each subset of samples used in the study

Category	Details	Screen		Cohort1		Cohort2		Combined cohort 1+2		NSCLC stage 1	Benign lung nodules
		NSCLC	Ctrl	NSCLC	Ctrl	NSCLC	Ctrl	NSCLC	Ctrl		
Number of samples		12	7	141	35	123	100	264	135	136	27
Age	Min	43	50	39	38	47	26	39	26	46	46
	Max	81	81	87	88	88	89	88	89	87	88
	Mean	65.8	66.6	66.2	63.8	69.2	57.6	67.6	59.2	68.8	62.0
	se	3.8	4.5	0.8	1.9	0.9	1.4	0.6	1.2	0.8	2.0
Gender	F	6	5	74	18	59	69	133	87	74	17
	M	6	2	67	17	64	31	131	48	62	10
Tobacco Use	Current	1	0	26	3	17	7	43	10	20	2
	past	9	6	103	26	91	38	194	64	98	20
	Never	2	1	12	6	15	52	27	58	18	5
	na	0	0	0	0	0	3	0	3	0	0
Histology	AC	8		99		81		180		97	
	LSCC	1		32		25		57		26	
	AC+LSCC	0		0		4		4		3	
	CARC	0		0		7		7		5	
	LCLC	0		0		1		1		0	
	NSCLC, NOS	3		10		5		15		5	
Stage	Stage I	4		72		64		136		136	
	Stage II	2		19		23		42		0	
	Stage III	6		43		31		74		0	
	Stage IV	0		7		5		12		0	

*AC = adenocarcinoma, LSCC = lung squamous cell carcinoma, CARC = carcinoid tumor, LCLC = large cell carcinoma, NSCLC, NOS-non small cell lung cancer, not otherwise specified

To further validate our finding we then assayed AKAP4 expression in a second independent cohort of patients collected at the Valley Hospital (VH), Paramus, N.J. This data set included 123 NSCLC patients and 100 controls (Table 1). The AUC of the ROC curve on the VH data set is 0.9805 (Figure 2B).

We then analyzed a combined data set that included the samples from both cohorts but not the discovery set. The combined data included 264 NSCLC patients and 135 controls. Using expression of AKAP4 as NSCLC classifier in the combined dataset the AUC of the ROC curve of 0.9714 (Figure 2C) with the distribution of expression values between NSCLC and control groups shown in Figure 2D. The AKAP4 expression level of −4.3 showed the most balanced sensitivity/specificity values (92.8% and 92.6% respectively) for the total accuracy of 92.7%. Linear discriminant analysis with cross-validation that included in addition to AKAP4 expression, age, gender and smoking status as potential predictors did not show an improved AUC or accuracy. The final observed performances of AKAP4 expression for classifications are summarized in Table 2. In addition, cross-validation studies were performed in order to estimate variations of reported AUC and accuracy values (Supplementary Table 1). Variation coefficient for the combined dataset was 0.10% for AUC and 0.16% for accuracy, indicating very high result confidence presented in the study.

**Table 2:** Performance of AKAP4 expression in the classification of different NSCLC and Control subsets

Comparison	ROC AUC[95% CI]	Sens	Spec	Acc
**Cohort 1** NSCLC *n* = 141 Controls *n* = 35	**0.9735** [0.9516,0.9953]	90.8%	100%	**92.6%**
**Cohort 2** NSCLC *n* = 123 Controls *n* = 100	**0.9805** [0.9669,0.9941]	95.1%	90.0%	**92.8%**
**Combined** NSCLC *n* = 264 Controls *n* = 135	**0.9714** [0.9563,0.9865]	92.8%	92.6%	**92.7%**
**Stage I VS all Controls** Stage I *n* = 136 Controls *n* = 135	**0.9795** [0.9650,0.9939]	93.4%	92.6%	**93.0%**
**NSCLC vs Nodules** NSCLC *n* = 264 Nodules *n* = 27	**0.9825** [0.9691,0.9958]	92.8%	100%	**93.5%**

For each classification the table lists observed AUC and 95% confidence interval (CI) and sensitivity (sens), specificity (spec) and overall accuracy (acc) for AKAP4 expression threshold of −4.3.

**Figure 3:**
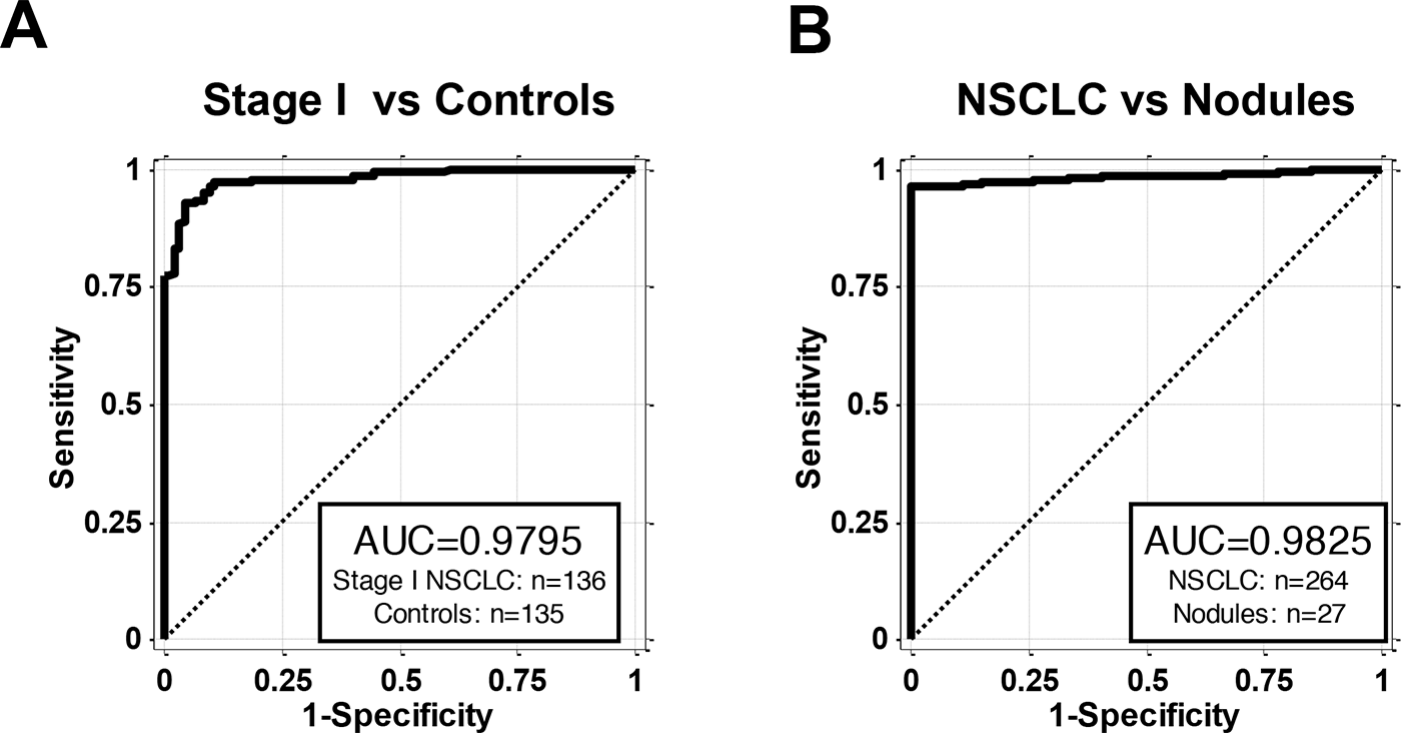
AKAP4 is a blood based biomarker for NSCLC early detection **A.** ROC curve of AKAP4 expression in the PBMC samples from 136 stage I NSCLC patients and 135 controls. AUC is 0.9795. **B.** ROC curve of AKAP4 expression in the PBMC samples from 264 NSCLC patients and 27 patients with benign lung nodules. AUC is 0.9825.

There were two samples subgroups of special interest: Stage I NSCLC, a group that benefits most from early detection when lung resection is most favorable and high risk lung nodules which require surgical confirmation to determine the malignant/non-malignant status. Among the 264 NSCLC patients, 136 were diagnosed as stage I NSCLC. AKAP4 expression levels used as a classifier for just the stage I NSCLC and all controls demonstrated the AUC of the ROC curve of 0.9795 (Figure 3A). Although the number of nodules included in this analysis is relatively small, the AUC of the ROC curve for classifying all 264 NSCLC vs 27 samples from patients with benign nodules gave a value of 0.9825 (Figure 3B), the best performance of all tested classifications, suggesting that AKAP4 expression associated with PBMC has the potential to distinguish benign from malignant lung nodules, a possibility that needs to be further addressed in a larger study.

### AKAP4 expression as a function of stage, cancer subtype, remission and recurrence

**Figure 4:**
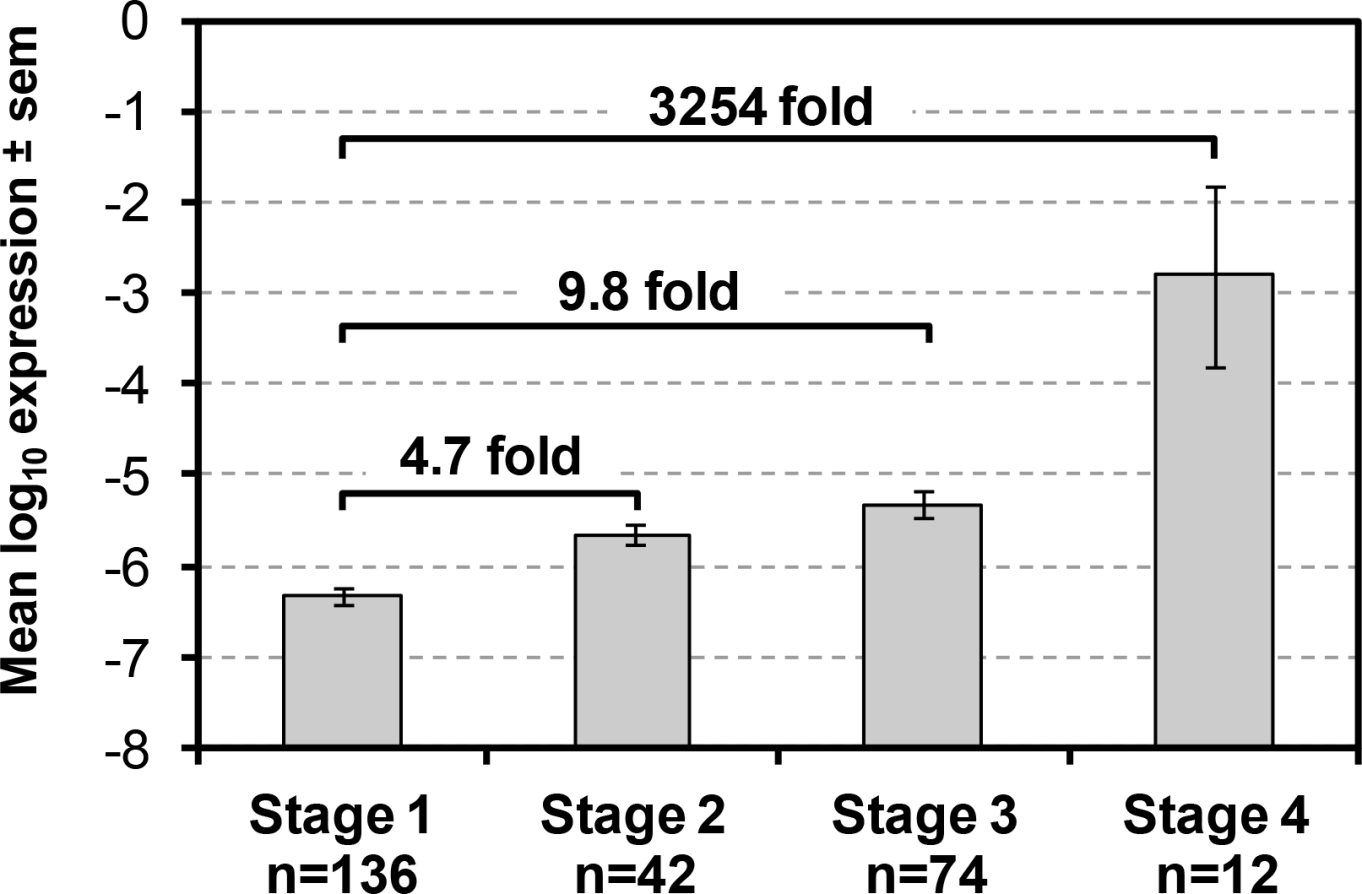
AKAP4 expression is associated with NSCLC stage Average AKAP4 expression ± standard error of mean is shown for each NSCLC Stage. Fold differences vs. Stage I are indicated.

Since in our previous PBMC gene expression study [18, 19] we found that patients with lung squamous cell carcinomas (LSCC) were more accurately classified than those with lung adenocarcinomas (AC) and that classification accuracy also increased with advanced cancer stages, we then tested whether the strength of AKAP4 PCR signal also correlated with a variety of clinical parameters including cancer stage and subtype. We performed linear regression analysis of AKAP4 expression using histology, stage, smoking history, gender and age as independent variables. The analysis identified that AKAP4 expression is significantly associated only with cancer stage (Supplementary Table 2). Indeed, Figure 4 shows that magnitude of AKAP4 expression consistently increases through all 4 stages, with the stage II, III and IV levels of AKAP4 being respectively on average 4.7, 9.8 and >3000 times the expression level in stage I. The fact that AKAP4 expression did not associate significantly with tobacco use also demonstrates that the AKAP4 classification may be used successfully for higher risk current smokers as well as lower risk non-smokers with the same efficiency.

**Figure 5:**
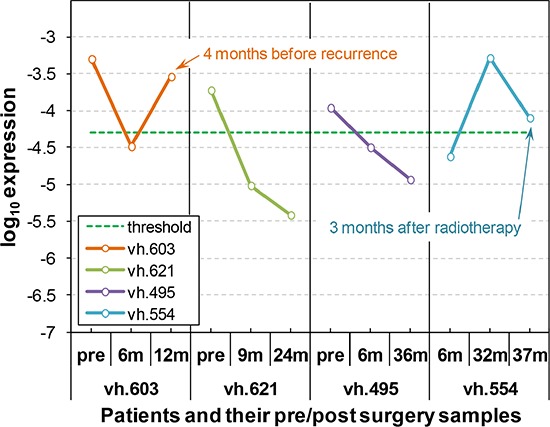
AKAP4 is a circulating biomarker for NSCLC disease monitoring and early detection of recurrence (patient vh.603) AKAP4 expression is determined in PBMC samples from a NSCLC patient at three time points: pre-surgery; 6 months post-surgery; and 12 months post-surgery. The AKAP4 expression is high before surgery but drops below the cutpoint 6 months post-surgery, indicating the patient is in remission. AKAP4 expression increased above the cutpoint by 12 months post-surgery, suggesting this patient had lung cancer. Approximately 4 months after the positive AKAP4 result, a second lung cancer nodule was detected by CT scan. (patient vh.621) AKAP4 expression is determined in PBMC samples from a NSCLC patient at three time points: pre-surgery; 9 months post-surgery; and 24 months post-surgery. The AKAP4 expression is high prior to surgery. The AKAP4 expression dropped below the cutpoint at 9 months post-surgery and stayed below the cutpoint 24 months post-surgery. Follow-up CT scans have not detected any lung nodules. This patient is currently in remission. (patient vh.495) AKAP4 expression is determined in PBMC samples from a NSCLC patient at three time points: pre-surgery; 9 months post-surgery; and 36 months post-surgery. The AKAP4 expression is high prior to surgery. The AKAP4 expression dropped below cutpoint 9 months post-surgery and stayed below cutpoint 36 months post-surgery. Follow-up CT scans have not detected any lung nodules. This patient is currently assessed as being in remission. (patient vh.554) AKAP4 expression is determined in PBMC samples from a NSCLC patient at three time points: 6 months post-surgery; 32 months post-surgery; and 37 months post-surgery. The AKAP4 expression was below cutpoint 6 months post-surgery, suggesting this patient is in remission. The AKAP4 expression increased above cutpoint 32 months post-surgery indicating a recurrence. CT scan and subsequent biopsy confirmed recurrent NSCLC. The AKAP4 expression decreased 3 months after radiation therapy but stayed above cutpoint, suggesting a residual cancer presence remained. This patient was diagnosed with metastatic lung cancer 10 months after radiation therapy.

We also explored whether PBMC associated AKAP4 expression changed after lung resection. We analyzed expression in samples from 4 NSCLC patients where follow up samples had been collected at several times after lung resection. We compared the AKAP4 expression in pre-surgery samples to expression at various times post-surgical resection. Four cases with somewhat different outcomes are shown in Figure 5. Outcomes for each case are described in detail in Supplementary Data. Patients vh.603, vh.623 and vh.495 show a significant drop in AKAP4 expression between the pre and post-surgery samples. AKAP4 expression for patients vh.623 and vh.495, who remained in remission 24 and 36 months post-surgery respectively remain below the cut-off value. Patient vh.603 had a sharp rise in AKAP4 expression 12 months post-surgery and 4 months prior to being diagnosed with a recurrence. The pre-surgery sample for patient vh.554 was not available but the 6 month post-surgery sample is in the non-cancer range. Patient vh.554 showed a sharp increase in AKAP4 at 32 months post-surgery associated with a diagnosed recurrence and underwent radiotherapy. The AKAP4 signal at the 3^rd^ time point, 3 months after radiotherapy has greatly decreased but remains in the cancer range.

## DISCUSSION

The global impact of lung cancer is strikingly evident in the million deaths that occur each year globally [1, 20, 21] with 64% of those deaths occurring in the economically developing world [1] where access to state of the art medical care such as LDCT is limited. While the National lung Screening Trial has demonstrated that a 20% reduction in lung cancer mortality is associated with routine LDCT screening of older individuals with a heavy smoking history, it also showed that for the majority of patients that had a positive screen for lung cancer based on lung nodules detected, approximately 96% of them proved to be false positives [3, 22]. These statistics highlight two unmet medical needs required to maximize the diagnostic potential of LDCT. The first is the development of diagnostic platforms that will distinguish nodules identified by routine LDCT screenings that are malignant from those that are benign. The second is the development of inexpensive, non-invasive methods that can identify at risk individuals who would benefit from follow up with LDCT. A variety of approaches focused on these difficult issues have been reported over the past several years and extensively reviewed recently [20]. They include several non-invasive procedures that identify panels of genes, proteins or antibodies whose expression is diagnostic for the presence of a NSCLC and include; bronchial brushings [23–25], saliva [26], PBMC [18, 27], plasma, or serum [28, 29] and whole blood [30] among others. Gene expression signatures have been identified ranging from 7 to 80 genes with AUCs of >80% [22] and with variable successes in distinguishing benign from malignant nodules. A recent proteomic study using a multiple reaction monitoring (MRM) assay has identified a 13 protein classifier in plasma that distinguishes benign from malignant nodules independent of their size with a negative predictive value of 90% [31]. Another recent study reports using antibodies present in serum to probe microarrays bearing thousands of random peptide sequences to identify disease specific hybridization signatures. They report a comparison between serum samples from lung cancer patients and sera of healthy controls with a reported accuracy for identifying lung cancers of 99% [32].

The presence of testis antigens, whose expression is primarily restricted to male germ cells, in a wide variety of cancers has generated a lot of interest in their utility as targets for therapy [10, 11, 33, 34]. Their potential utility as blood based cancer biomarkers because of their expression on CTCs or cancer derived exosomes [35–37] has been less well studied. Our results indicate that their expression in blood cells can be used as cancer biomarkers. Further studies in this aspect are needed to explore the potential application of their expression in cancer detection.

To determine whether PBMC associated CTA expression could be diagnostic for NSCLC we first carried out a pilot study on PBMC derived RNA from 12 NSCLCs and 7 controls and assayed for expression of 116 CTAs using nested PCR in order to find the best potential candidates for further analyses on a large data set. We identified several potential candidates that distinguished cancers and controls but only 2 showed exceptional accuracy, AKAP4 and GAGE 4. Of the 2, AKAP4 was the most accurate and GAGE4 was later eliminated because of poor performance when applied to the larger data set. AKAP4 is a known cancer/testis gene located on the X chromosome and has been shown to be aberrantly expressed in a variety of different cancers [38–40] as well as on circulating tumor cells (CTCs) [40].

AKAP4 is a member of the A-kinase anchor proteins which bind the Protein kinase A (PKA) regulatory subunit and functions to anchor PKA to specific cellular locations. It has been identified as a tumor antigen, and as a potential therapeutic target for cervical and ovarian cancer [38, 39], multiple myeloma [41], breast cancer [42], prostate cancers [43] and importantly for NSCLCs [40]. Because expression of AKAP4 is normally confined to testis (33) the background expression in cancer free controls is essentially negative. While the detection of this message in PBMC samples raises the possibility expression is associated with CTCs, CTC numbers, especially in our early stage samples are expected to be quite low. Another potential source of the AKAP4 signal are tumor derived exosomes which are released in large numbers and engulfed by tumor infiltrating lymphocytes including macrophages that are included in the PBMC fraction [36, 37, 44, 45]. A third alternative that low level expression is induced in specific immune cells in the PBMC fraction by the presence of a tumor in the lung cannot be eliminated, but seems less likely.

That the origin of the AKAP4 signal we detect is the lung cancer is further supported by the strong correlation of signal intensity to tumor stage as shown in Figure 4. No other patient characteristic including age, gender, tobacco use or cancer subtype showed any significant correlation. The tumor derivation of this signal is further supported by data demonstrating that the AKAP4 presence in PBMC RNA is significantly reduced after successful lung resection and that expression increases once again with a lung tumor recurrence. Although the number of post resection patients analyzed is small, the results from different post resection time points that correlate with either remission or recurrence are compelling and worthy of further examination. Taken together these observations support a strong relationship between the presence of a tumor in the lung and the detection of AKAP4 message in the peripheral blood samples.

The 141 NSCLC samples in our first validation set had been previously analyzed using a 29 gene mRNA classifier identified by microarrays. This signature did not include AKAP4 (18). This is not surprising as our reanalysis of this data shows that AKAP4 expression levels are too low to be reliably detected by microarray technology. Eight of those samples (3 stage 1, 4 stage 2, and 1 stage 3) in this study were incorrectly classified as controls by AKAP4 levels. These samples were previously analyzed using our 29 gene microarray signature [18] with 7 of the 8 cancers being correctly classified in that study, suggesting the two signatures are detecting different tumor related/initiated processes.

Further development and refinement of this test and validation in larger numbers of samples are in progress. The combination of this test with imaging techniques such as LDCT or with a variety of other methods that have been described has the potential to dramatically improve the sensitivity and specificity of lung cancer diagnosis, to assist in clinical evaluations of recurrence, and ultimately to improve patient outcomes. The patients with benign nodules in data set 1 could have been spared their lung resections if a robust, accurate and rapid alternative test was available. While our study on remission and recurrence samples is small, the results are very encouraging that AKAP4 may potentially provide a sensitive marker to track remission and act as an early marker of recurrence. Although further validation is required, we describe a simple test that has the potential to contribute to the development of more informed approaches to individual patient care.

## MATERIALS AND METHODS

### Patients and controls

The discovery set of 12 cancers and 7 control samples used for the initial biomarker selection and the 141 cancers and 35 controls in the first validation cohort were previously described as a part of a larger sample set [18, 19]. A second cohort of 123 cancers and 100 controls were collected at The Valley Hospital, One Valley Health Plaza, Paramus, NJ 07652 for a total of 264 confirmed NSCLCs and 135 controls. The demographics for all the samples sets used in the study are summarized in Table 1.

### PBMC collection

BD Vacutainer CPT Tubes were used for blood collection and PBMC were isolated according to the manufacturer's directions within 3 hours of collection and maintained at −80°C until RNA extraction.

### Total RNA Isolation

RNA was extracted from normal and lung cancer PBMC pellets using TRI Reagent (T9424 Sigma-Aldrich) as previously described [18]. Briefly, 1 ml of TRI Reagent was added to the cell pellet and incubated for 15 min at room temperature. 1 μl of linear acrylamide was added and incubated further for 5 min at room temperature. Then 100 μl of 1-bromo-3-chloropropane was added, vortexed for 30 seconds, incubated for 10 minutes at room temperature followed by centrifugation at 12000g for 15 minutes at 4°C. The aqueous phase was collected; 1 μl of RNAsin (N2115 Promega) and 500ul of isopropanol were added. After 10 minutes incubation at room temperature samples were centrifuged at 12000g for 10 minutes at 4°C. The RNA pellet was washed twice with 1ml of 75% ethanol. The pellet was air dried, and suspended in 50 μl of RNAse free water and stored at −80°C. RNA concentration and quality was assessed using the NanoDrop.

### Reverse transcription

cDNA was synthesized from total RNA using the High Capacity cDNA Reverse Transcription Kit (Applied Biosystems, P/N 4374966) following the manufacturer's instructions. Briefly, 250 ng of total RNA was used as template in a total volume of 20 μl containing 2 μl 10X RT buffer, 0.8 μl 100 mM dNTP mix, 2 μl 10X RT Random Primers, 1 μl Multiscribe Reverse Transcriptase, 1 μl RNase Inhibitor. The reactions were incubated at 25°C for 10 min, 37°C for 2 hrs and 85°C for 5 min. cDNA samples were stored at −20°C.

### Quantitative real time PCR

The reference and genomic sequence for each gene included in the study was obtained from the UCSC Genome Bioinformatics website. RT-PCR primers were designed using Primer Express (Applied Biosystems). Gene specific primers were designed manually. Because the genes of interest were expressed at low levels in PBMC RNA, nested PCR was used to quantitate gene expression. The first round of PCR was carried out in 50 μl reaction containing 5 μl of cDNA, 10 μl 10X buffer, 1 μl 10 mM dNTP, 3 μl gene specific primer mix (20 μM), 5 units Taq Polymerase and amplified on a thermal cycler at 94°C for 2minutes followed by 40 cycles of 94°C for 30 seconds, 60°C for 1 min and 72°C for 15 seconds, then 1 cycle of 72°C for 10 min, ending at 4°C. The PCR primers for the 1^st^ round amplification of AKAP4 are: 5′ TCCTACATGATGGCGTACTCTG and 5′ AAGTTGCCTTCTGAGCTGGAAC; and GAGE4 are 5′CCAGGGAGCTGTGAGGCAG and 5′ ACACCCAGTCTGTGGGTGAC. Real time PCR reactions were performed to detect the expression of each gene in duplicate, in 25 μl reaction volume using 5 μl of 1^st^ round PCR product, 12.5 μl SYBR Select Master Mix (Applied Biosystems), 0.25 μl primer mix (2 μM final) and 7.25 μl water. Ribosomal 5S gene is a housekeeping gene and was used for normalization. The real time PCR primers for AKAP4 are: 5′ GGGTGTGTGCAAGGTAGATCTCT and 5′ CACATCGACAAAGCATATCACTTTC. The GAGE4 primers are: 5′ GCTGTGAGGCAGTGCTGTGT and 5′ TTCCTCGCCAACTCATATTTCA. The real time PCR primers for 5S are: 5′ GCCATACCACCCTGAACG and 5′ AGCCTACAGCACCCGGTATT. As a negative control, wells without any template were also assayed. All reactions were carried out on the 7500 Fast Real Time PCR system (Applied Biosystem). The average Ct (threshold cycle) of duplicate assays for each gene and sample was calculated. Expression values for AKAP4 or GAGE4 were calculated using difference in Ct values vs the endogenous reference control gene 5S as 2−Δ^Ct^, where ΔCt = Ct_gene_–Ct_5S_. then log_10_-transformed (values <10^−7^ were assigned a value of 10^−7^ to allow log-transformation of zero values).

### Statistical analysis

For each cohort of samples, the selected biomarker AKAP4 or GAGE4 was examined as the classifier of a test for the presence of lung cancer. First, a nonparametric receiver operating characteristic (ROC) curve was plotted and the area under the ROC curve (AUC) and its 95% confidence interval were estimated [46]. AUC can be interpreted as the probability that the result of a diagnostic test of a randomly selected abnormal subject will be greater than the result of the same diagnostic test from a randomly selected normal subject. The greater the AUC, the better the global performance of the diagnostic test is with a maximum of 1 indicating a perfect classifier. To examine the reliability of classification with AKAP4, we also performed linear discriminant analysis using AKAP4 expression levels as the predictor with 1000 times of 10-fold cross-validations. The averages of AUC and accuracy with their standard errors were estimated from these cross-validation steps and variation coefficients for all subsets were calculated to demonstrate relative variability of presented performance values. Accuracy, sensitivity and specificity for all original data sets and sample designation for pre/post-surgery samples were evaluated using the “cutpoint” obtained from the data for the combined samples from cohorts 1 and 2. The cutpoint for the AKAP4 expression threshold of −4.3 shown in Figure 2D was chosen based on the combined dataset as a cutpoint with the most equal sensitivity and specificity (92.80% and 92.59% respectively). Samples with AKAP4 expression values above this cutpoint were designated as cancer and samples with values below as non-cancer.

## SUPPLEMENTARY DATA FIGURE AND TABLES


