# Supplementary material for: AKAP4 is a circulating biomarker for non-small cell lung cancer
Source: Oncotarget. 2015 May 13;6(19):17637–47. doi: 10.18632/oncotarget.3946 (PMC4627334; doi:10.18632/oncotarget.3946)
Supplement: Supplementary file 1 [file oncotarget-06-17637-s001.pdf]

SUPPLEMENTARY DATA

Case 1 – Recurrence

Patient vh.603 is an 85 year-old female ex-smoker diagnosed as stage I NSCLC who underwent lung resection. CT scans were assessed in the follow-ups and PBMC samples were collected at 3 time points from this patient: pre-surgery, 6 months post-surgery, and 12 months post-surgery. Approximately 16 months after surgery, a lung nodule was found by CT scan and subsequently confirmed as NSCLC. We carried out nested PCR on all available samples and used the cutpoint determined on the large dataset to assess AKAP4 expression. As expected the AKAP4 expression in the pre-surgery sample is above the cutpoint, confirming the presence of NSCLC in agreement with the clinical diagnosis (Figure 5). The AKAP4 expression drops to below the cutpoint in the 6 months post-surgery sample, indicating that the patient was in remission at 6 months (Figure 5) based on our case control study. Expression subsequently increased to above cutpoint in the 12 months post-surgery sample, suggesting the cancer had recurred (Figure 5) although no evidence of recurrence was detected by CT scan at this time. Approximately 4 months later (16 months post-surgery), a lung nodule was detected by CT scan and subsequently confirmed as NSCLC.

Case 2 – Remission

Patient vh.621 is an 85 year-old female ex-smoker, diagnosed with Stage I NSCLC, her tumor was removed by surgery and the patient is currently diagnosed as in remission. PBMC samples were collected at 3 time points: pre-surgery, 9 months post-surgery, and 24 months post-surgery. AKAP4 expression in the pre-surgery sample is above the cutpoint, consistent with the presence of a NSCLC (Figure 5). The AKAP4 expression dropped below the cutpoint at 9 months post-surgery and remained below the cutpoint 24 months post-surgery (Figure 5). By clinical assessment this patient remains in remission.

Case 3 – Remission

Patient vh.495 is a 67 year-old female ex-smoker also diagnosed as stage I NSCLC. PBMC were collected at 3 time points from this patient: pre-surgery, 9 months post-surgery, and 36 months post-surgery. The AKAP4 expression dropped from above the cutpoint before surgery to below the cutpoint 9 months after surgery and remained below the cutpoint at 36 months post-surgery (Figure 5). Repetitive CT scans confirm that this patient is currently in remission.

Case 4 – Recurrence + treatment

We also determined AKAP4 expression in PBMC samples from a patient who relapsed and subsequently underwent additional treatment. Patient vh.554 is a 79 year-old female current smoker diagnosed as stage I NSCLC. Surgery was performed to remove her tumor and 32 months later, a lung nodule was detected by CT scans during follow-up. The nodule was confirmed as NSCLC by biopsy. This patient underwent stereotactic body radiation therapy. CT scans after radiation therapy did not detect the presence of any lung nodule. However, metastatic disease was detected 12 months after the finding of the second tumor. We analyzed AKAP4 expression in this patient blood at 3 time points from this patient: 6 month post-surgery, 32 months post-surgery, which was at the time of recurrence detection but pre-radiation therapy, and 37 months post-surgery (3 months post-radiation). A pre-surgery blood sample was not available. While the AKAP4 expression was below the cutpoint 6 months post-surgery (Figure 5), expression was increased significantly above the cutpoint 32 months post-surgery supporting the CT and biopsy diagnosis of a NSCLC recurrence. At 37 months post-surgery (3 months post-radiation therapy) AKAP4 levels decreased from pre-radiation therapy values but remained above the cutpoint, suggesting a residual cancer was present even though CT scan did not detect any lung nodule at this time and there was no clinical indication of residual disease. This patient was clinically diagnosed as metastatic NSCLC 10 months post-radiotherapy. These results from an admittedly small sample size support the potential utility of monitoring AKAP4 expression as a marker of remission/recurrence even in the absence of a positive CT scan.

SUPPLEMENTARY FIGURE AND TABLES

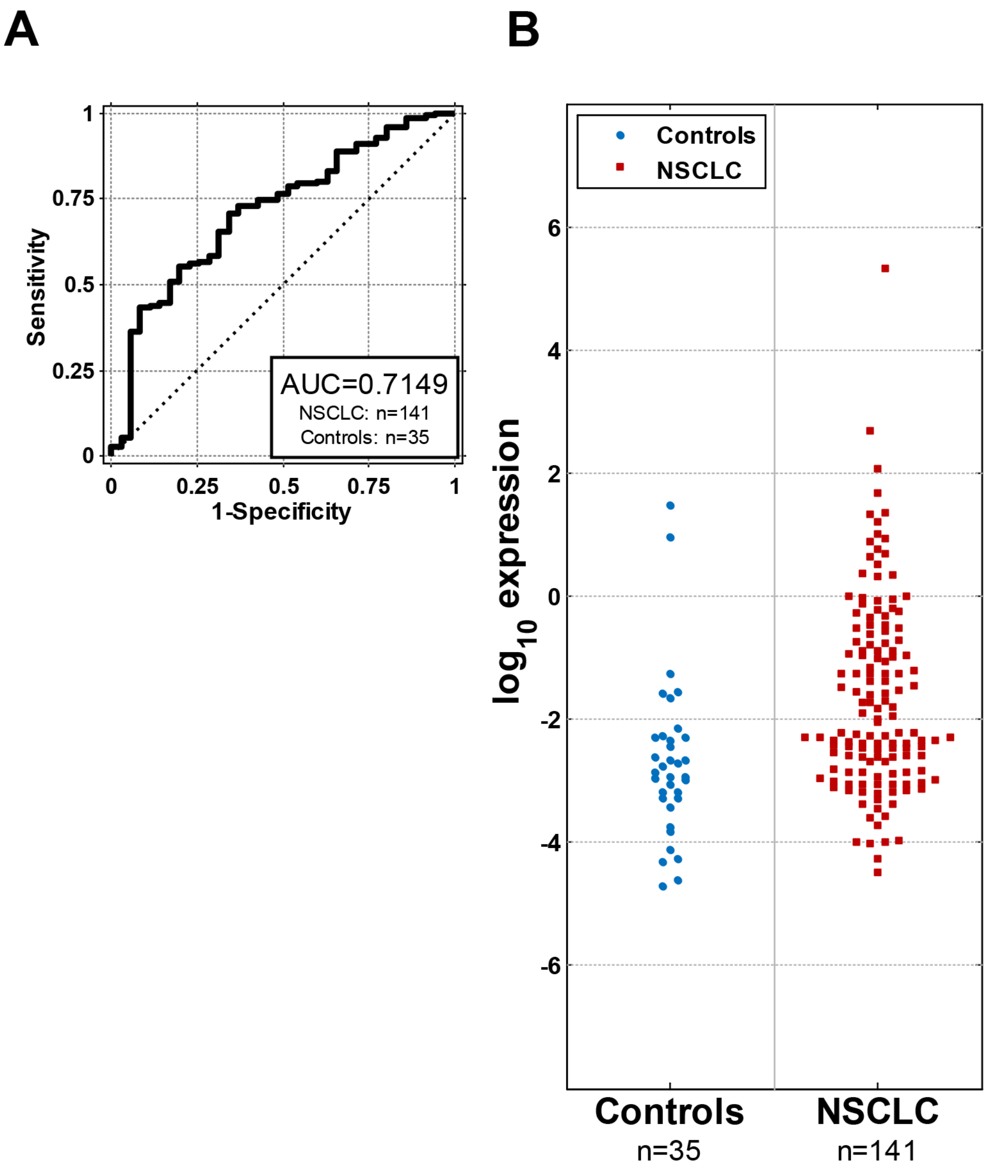

Supplementary Figure 1: GAGE4 expression performance in classification of samples from cohort 1. A. ROC curve; **B.** expression levels for individual samples.

Supplementary Table 1: Mean, standard error (se) and variation coefficient (var = se/mean) for AUC and accuracy estimated from cross-validation analysis

Classification	Cross-validation values					
	AUC			Accuracy		
	mean	se	var	mean	se	var
**Cohort 1** NSCLC $n = 141$ Controls $n = 35$	0.9680	0.0031	0.32%	94.82%	0.26%	0.27%
**Cohort 2** NSCLC $n = 123$ Controls $n = 100$	0.9795	0.0008	0.08%	90.88%	0.29%	0.32%
**Combined** NSCLC $n = 264$ Controls $n = 135$	0.9699	0.0010	0.10%	91.88%	0.15%	0.16%
**Stage I vs all Controls** Stage I $n = 136$ Controls $n = 135$	0.9771	0.0012	0.12%	88.88%	0.27%	0.30%
**NSCLC vs Nodules** NSCLC $n = 264$ Nodules $n = 27$	0.9810	0.0011	0.11%	90.72%	0.00%	0.00%

Supplementary Table 2: Results of linear regression analysis of AKAP4 expression and different clinical parameters. Results demonstrate that only Stage was significantly associated with AKAP4 expression.

Variable	Significance ($p < 0.05$)	beta value	95% CI
**AC diagnosis**	ns	–0.045	[–0.581, 0.491]
**LSCC diagnosis**	ns	–0.01	[–0.635, 0.614]
**Stage**	**Significant**	**0.214**	**[0.049, 0.379]**
**Never smokers**	ns	–0.182	[–0.822, 0.458]
**Current smokers**	ns	–0.008	[–0.446, 0.431]
**Years of smoking**	ns	–0.009	[–0.020, 0.001]
**Packs per year**	ns	–0.051	[–0.284, 0.182]
**Gender**	ns	–0.028	[–0.355, 0.298]
**Age**	ns	–0.012	[–0.029, 0.005]
